# A meta-analysis comparing the efficacy of mineralized collagen-polymethylmethacrylate and polymethylmethacrylate bone cements in the treatment of vertebral compression fractures

**DOI:** 10.1371/journal.pone.0299325

**Published:** 2024-03-08

**Authors:** Song-feng Li, Xi-yong Li, Xiao-hui Bai, Yun-lu Wang, Peng-fei Han, Hong-zhuo Li

**Affiliations:** 1 Department of Orthopaedics, Heping Hospital Affiliated to Changzhi Medical College, Changzhi, P.R. China; 2 Graduate School, Changzhi Medical College, Changzhi, P.R. China; American Dental Association Science & Research Institute (ADASRI), UNITED STATES

## Abstract

**Purpose:**

Vertebral compression fractures are often treated with vertebroplasty, and filling the injured vertebrae with bone cement is a key part of vertebroplasty. This meta-analysis was performed to compare the clinical efficacy and safety of mineralized collagen—polymethylmethacrylate (MC-PMMA) and polymethylmethacrylate (PMMA) bone cement in the treatment of vertebral compression fractures by vertebroplasty.

**Methods:**

A computerized search of the published literature on mineralized collagen-polymethylmethacrylate and polymethylmethacrylate bone cement in the treatment of vertebral compression fractures was conducted in the China National Knowledge Infrastructure (CNKI), Wanfang database, PubMed, Embase, and Cochrane Library. The search was carried out from the time the database was created to March 2023 and 2 researchers independently conducted literature searches to retrieve a total of 884 studies, of which 12 were included in this meta-analysis. Cochrane systematic review methods were used to assess the quality of the literature and a meta-analysis was performed using ReviewManager 5.4 software.

**Results:**

The results of the present meta-analysis showed that in postoperative adjacent vertebral fractures [OR = 0.25; 95% CI (0.15, 0.41)], postoperative cement leakage [OR = 0.45; 95% CI (0.30, 0.68)], Oswestry Disability Index (ODI) scores in the first 3 days after surgery [OR = -0.22; 95% CI (-0.42, -0.03)], ODI score at 6–12 months postoperatively [OR = -0.65; 95% CI (-0.97, -0.32)], visual analog scale (VAS) score at 6–12 months postoperatively [OR = -0.21; 95% CI (-0.46, 0.04)], and 1-year postoperative CT values [OR = 5.56; 95% CI (3.06, 8.06)], the MC-PMMA bone cement group was superior to the PMMA bone cement group. However, the differences between the two groups were not statistically different in terms of cement filling time, cement filling volume, operation time, intraoperative bleeding, hospitalization time, postoperative (<1 week, 3–6 months) vertebral body posterior convexity Cobb’s angle, postoperative (<1 week, 6–12 months) vertebral body anterior margin relative height, postoperative (≤3 days, 1–3 months) pain VAS score and postoperative (1–3 months) ODI score.

**Conclusions:**

Compared with PMMA bone cement, the application of MC-PMMA bone cement is advantageous in reducing postoperative complications (adjacent vertebral fracture rate, cement leakage rate), pain relief, and functional recovery in the long-term postoperative period (>6 months), but there is still a need for more high-quality randomized controlled studies to provide more adequate evidence.

## 1. Introduction

Vertebroplasty (VP) is the most widely used minimally invasive technique for the treatment of vertebral compression fractures, which has the advantages of simple operation, less invasiveness, rapid analgesia, and rapid rehabilitation [1]. Currently, two minimally invasive surgical procedures are commonly used, including percutaneous vertebroplasty (PVP) and vertebral kyphoplasty (PKP). As once a vertebra is fractured, patients often experience severe pain response and limited spinal mobility, requiring long-term bed-resting treatment, leading to a significant decrease in quality of life [2]. It has been shown that VP can rapidly strengthen the vertebral body by filling the fractured vertebrae with bone cement to achieve mechanical stability of the injured vertebrae, thereby relieving the pain symptoms and improving the limitation of physical activities in patients with vertebral fractures [3].

In the 1880s, the French company Deramond first applied VP technology to fill bone cement into the vertebral body of a patient with vertebral hemangioma with success [4]. Currently, commonly used bone cement materials in VP mainly include injectable polymethylmethacrylate (PMMA), biodegradable bone cement, calcium phosphate bone cement (CPC), and composite bone cement [2]. Different filling materials have different effects on the biomechanical properties of the vertebral body, thus producing different biomechanical effects on neighboring vertebrae. Therefore, the choice of filling materials for injured vertebrae becomes one of the key factors affecting clinical outcomes.

One of the main ingredients of PMMA is a self-curing acrylic compound. Initially used in prosthetic replacement and dentistry, PMMA was reported to be first successfully applied by Charnley in hip replacement, and then gradually applied in various types of orthopedic surgeries [2, 5]. PMMA has become the most widely used filler material in VP because of its injectability, short self-curing time, high adhesion, good mechanical properties, and rapid pain relief [6]. However, with the clinical application of PMMA, some drawbacks have been exposed, such as the inability of the filler material to be degraded, low biocompatibility, damage to the surrounding tissues due to exothermic polymerization, low viscosity of the bone cement that can easily lead to damage to the spinal cord or nerves from leakage of the bone cement [7], and excessive enhancement of the mechanical strength of the vertebral body leading to an excessively high modulus of elasticity of the solidified body (up to 2~3 GPa), which is much higher than the modulus of elasticity of human cancellous bone (0.05~0.8 GPa), which makes it easy to wear through the endplates and lead to fractures of neighboring vertebrae, etc. [8].

To improve the shortcomings of PMMA such as excessive hardness and poor biocompatibility, a novel bone cement material with mineralized collagen-modified PMMA has been developed and applied in VP. Mineralized collagen (MC) consists of collagen and hydroxyapatite, which is assembled by nano-calcium, phosphorus salts, and collagen molecules using a unique in vitro biomimetic mineralization technique, and its chemical composition and structure are similar to that of natural human cancellous bone [9]. It has been confirmed that mineralized collagen artificial bone repair materials not only have excellent osteoinductive properties, but also can eventually be completely degraded and resorbed in vivo [10, 11], and are widely used in clinical applications. MC particles were added to PMMA bone cement to obtain a new type of bone cement with the advantages of both PMMA and MC. Kong et al. [12] found that the incorporation of MC into biologically inert PMMA was beneficial for improving its biocompatibility and inducing interaction between the filler material and the host bone tissue through an in vivo study in animals. This suggests that MC-PMMA composite filler materials have practical clinical applications.

MC-PMMA and PMMA are commonly used filler materials in VP, but the clinical efficacy of the two is still controversial, and there is no meta-analysis comparing the clinical efficacy of MC-PMMA and PMMA bone cement in repairing injured vertebrae. Therefore, the purpose of this meta-analysis is to compare and evaluate the postoperative efficacy and clinical application of MC-PMMA and PMMA bone cement in the treatment of vertebral compression fractures in patients with vertebral body compression fractures according to the current published literature.

## 2. Materials and methods

### 2.1 Normal information

The study population included published clinical controlled studies. Patients were identified for vertebroplasty (both PVP and PKP) based on patient history, physical examination, and imaging. Non-case control studies, case reports, review literature, correspondence, and duplicate reports were excluded. The intervention was MC-PMMA with PMMA bone cement. The primary outcome indicators were postoperative pain VAS score, postoperative ODI score, and postoperative complications (adjacent vertebral fracture, and cement leakage rate); The secondary outcome indicators were the postoperative vertebral kyphosis Cobb angle, the postoperative CT value, the relative height of the anterior margin of the vertebrae, the amount of cement filling, the time of cement filling, the time of surgery, the intraoperative bleeding, and the length of hospital stay, for a total of 11 items.

### 2.2 Search strategy

Among the databases searched were Pubmed, Embase, Cochrane Library, CNKI, and Wanfang database. Tables of contents and citation tables were manually searched to locate grey literature such as unpublished academic papers, chapters in monographs, etc. All relevant literature was also searched without restriction on language. The English search terms are Mineralized collagen-Polymethylmethacrylate, MC-PMMA, Polymethylmethacrylate, PMMA, Vertebroplasty, and Percutaneous vertebroplasty. PVP, Percutaneous Kyphoplasty, PKP, Spine. The literature search was carried out independently by two searchers who cross-checked the results.

### 2.3 Quality assessment of the literature

The included literature was analyzed independently by two physicians according to appropriate evaluation criteria, and in case of disagreement, it was discussed and resolved or referred to a third senior physician to jointly adjudicate the quality of the literature. The risk of Cochrane bias was assessed strictly according to the following criteria: (I) generation of randomized sequences; (II) whether the double-blind principle was used for subjects and investigators; (III) completeness of experimental data; (IV) whether allocation concealment was used; (V) whether the experiment was selective for study outcomes; and (VI) other biases. The quality of the literature was also evaluated according to the Newcastle-Ottawa scale (NOS), which focuses on 3 aspects of study population selection, comparability between groups, and outcome measures, with a total score of 9. A total literature score >7 is considered high-quality case study literature.

### 2.4 Statistical analysis

Data were analyzed using ReviewManager 5.4 software (https://www.cochrane.org/) provided by the Cochrane Collaboration. Dichotomous variables were expressed as odds ratio (OR) and 95% confidence interval (CI), and continuous variables were expressed as mean difference (MD) or standardized mean difference (SMD) and 95% CI. The I^2^ value was calculated to test the heterogeneity among different studies, and if the heterogeneity among studies was small (I^2^ < 50% and P > 0.1), a fixed effect model (fixed effect) was used; whereas, if the heterogeneity among studies was large (I^2^ ≥ 50% and P ≤ 0.1) and a random effect model (random effect) was used, otherwise, after excluding obvious sources of heterogeneity, a fixed effect model (fixed effect) was used to analyze the results. For the sources of heterogeneity, a fixed-effects model was used for the analysis. The sensitivity analysis was performed by applying a one-by-one exclusion of literature and re-analysis. P < 0.05 was considered statistically significant when the difference was.

## 3. Results

### 3.1 Search results

**Fig 1:**
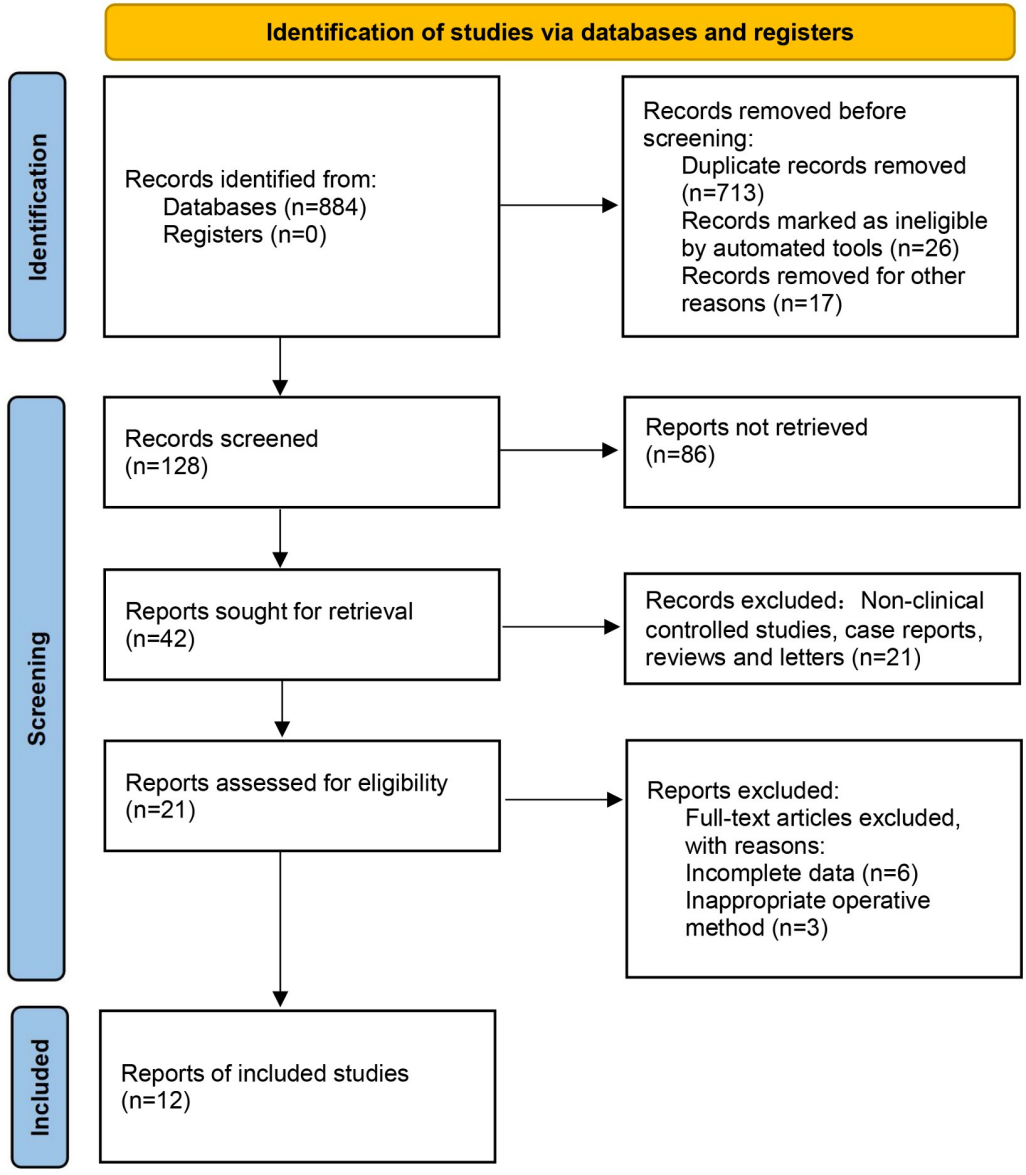
Flow diagram of study identification and selection.

Based on the above search strategy, a total of 884 relevant documents were retrieved. According to the inclusion and exclusion criteria, 128 relevant papers were initially screened by reading the titles and abstracts and excluding 756 papers that did not match; then, 12 papers [13–24] were finally included in the analysis by reading the full text. Baseline conditions such as patient age and disease duration were analyzed in each included literature and were comparable (P>0.05). The specific literature search and screening process and results are shown in Fig 1, and the basic characteristics of the included literature studies are shown in Table 1.

**Table 1:** Baseline characteristics of the studies included in the meta-analysis.

Author	Study Design	Surgical methods	Year	Group	Patients	Age (years)	Mineralized collagen by weight percentage(%)	Gender (M/F)	Outcomes	Newcastle-Ottawa scale
Bai [13]	RCT	PKP	2017	MC-PMMA	48	55.3±7.1	14.29%	22/26	(4)(10)(11)	7
				PMMA	47	56.2±6.1	-	20/27		
Luo [14]	Retrospective	PVP	2020	MC-PMMA	31	83.3±2.9	13.08%	6/25	(1)(3)(7)(9)	7
				PMMA	32	84.5±3.5	-	5/27		
Tang [15]	Prospective	PKP	2021	MC-PMMA	14	65.7±11.9	NA	6/8	(1)(2)(4)(7)(9)(10)(11)	8
				PMMA	14	68.2±8.5	-	5/9		
Wang [16]	Retrospective	PVP	2018	MC-PMMA	50	72.2±5.9	15.00%	6/44	(6)(7)(8)(9)(11)	7
				PMMA	30	72.7±6.2	-	8/22		
Zhu [17]	Retrospective	PKP	2018	MC-PMMA	46	72.3±11.2	14.29%	14/32	(1)(5)(8)(9)(10)(11)	7
				PMMA	48	72.6±9.9	-	13/35		
Zhu [18]	RCT	PKP	2020	MC-PMMA	12	74.1±9.3	15.00%	9/3	(9)(10)(11)	7
				PMMA	12	75.3±8.8	-	7/5		
Zhu [19]	Retrospective	PVP	2021	MC-PMMA	40	71.2±11.2	15.00%	12/28	(1)(2)(3)(6)(7)(9)	7
				PMMA	39	71.5±7.9	-	13/26		
Chen [20]	RCT	PKP	2021	MC-PMMA	60	75.5±7.7	NA	20/40	(4)(9)(10)(11)	8
				PMMA	60	74.8±7.5	-	22/38		
Huang [21]	Prospective	PVP	2021	MC-PMMA	65	53.9±9.8	16.67%	37/28	(1)(2)(3)(5)(9)(10)(11)	7
				PMMA	58	54.8±9.1	-	33/25		
Jiang [22]	RCT	PVP	2020	MC-PMMA	30	62.5±4.9	NA	12/18	(4)(5)(9)(10)(11)	7
				PMMA	30	63.2±4.9	-	10/20		
Luo [23]	Retrospective	PKP	2018	MC-PMMA	9	73.6±5.7	15.00%	2/7	(1)(2)(3)(9)(10)(11)	6
				PMMA	14	70.0±8.6	-	2/12		
Meng [24]	RCT	PKP	2021	MC-PMMA	166	70.8±9.0	14.29%	40/126	(1)(5)(6)(7)(9)(10)(11)	8
				PMMA	148	69.9±7.8	-	31/117		

RCT: Randomized Controlled Trial; MC-PMMA: Mineralized collagen-polymethylmethacrylate; PMMA:polymethylmethacrylate; PVP: Percutaneous vertebroplasty; PKP: Percutaneous Kyphoplasty; NA: Not Available. (1)Operative time (min) (2)Intraoperative bleeding (ml) (3)Length of hospital stay (day) (4)Postoperative posterior vertebral body convexity Cobb angle (5)Postoperative anterior vertebral body margin relative height (6)Cement filling volume (ml) (7)Cement filling time (min) (8)Postoperative CT value (9)Postoperative complications (adjacent vertebral body fracture, cement leakage rate) (10)Postoperative ODI score (11)Postoperative pain VAS score.

### 3.2 Quality evaluation of the included studies

**Fig 2:**
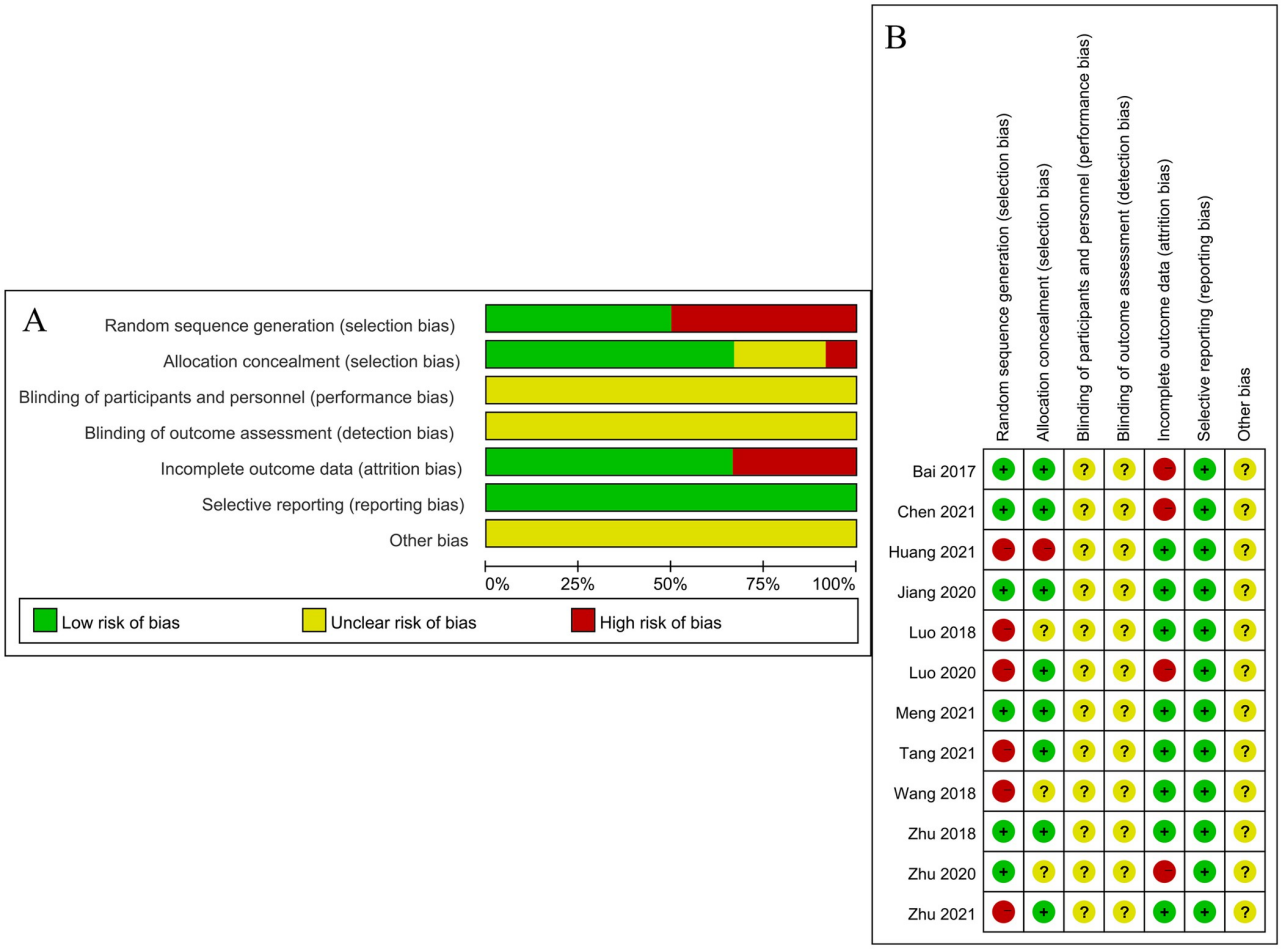
Summary of risk of bias. (A) Risk of bias graph. (B) Risk of bias. Green: low risk; Red: high risk; Yellow: not mentioned in the article.

Nine papers were included in this study, two prospective, five retrospective, and five randomized controlled studies. The Newcastle-Ottawa scale (NOS) was used for quality assessment. Of those, three trials scored an 8, eight a 7 and one a 6. Despite the limited amount of literature included in this study, the overall quality of the literature is high and the results of the specific literature quality assessment are shown in Fig 2.

### 3.3 Meta-analysis results

#### 3.3.1 Comparison of postoperative pain VAS scores (≤3 days, 1–3 months, 6–12 months)

**Fig 3:**
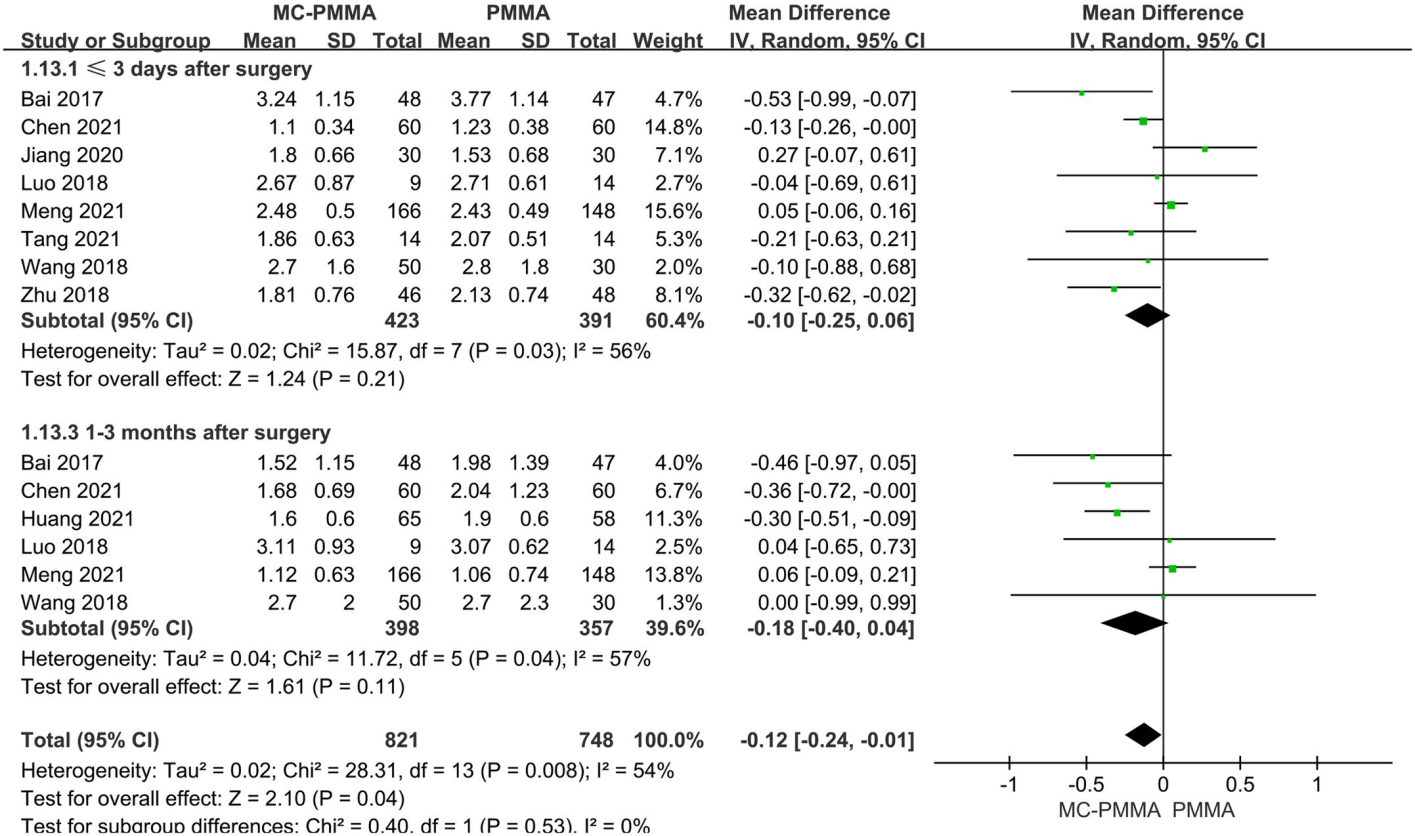
Forest plots of studies assessing pain VAS scores in the MC-PMMA vs. the PMMA group at postoperative (≤3 days, 1–3 months).

**Fig 4:**
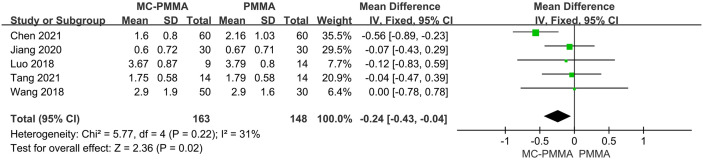
Forest plots of studies assessing pain VAS scores in the MC-PMMA vs. the PMMA group at postoperative (6–12 months). The positive effect represents better postoperative pain relief in patients with MC-PMMA bone cement after vertebroplasty, and the negative effect represents better postoperative pain relief in patients with PMMA bone cement after vertebroplasty.

Eight of the 12 studies reported visual analogical scale (VAS) scores of pain within 3 days postoperatively between the two groups and included 814 patients. The results of the heterogeneity test between studies showed the presence of heterogeneity (I^2^ = 56%; Q-test, P = 0.03), so Meta-analysis was performed using a random-effects model, and the results showed no statistical significance (Z = 1.24; P = 0.21; OR = -0.10; 95% CI (-0.25, 0.06)) (Fig 3). Six studies reported pain VAS scores at 1–3 months postoperatively between the two groups and included 755 patients. Heterogeneity existed between studies (I^2^ = 57%; Q-test, P = 0.04), so Meta-analysis was performed using a random-effects model, which showed no statistical significance between the two groups (Z = 1.61; P = 0.11; OR = -0.18; 95% CI (-0.40, 0.04)) (Fig 3). Five studies reported pain VAS scores at 6–12 months postoperatively between the two groups and included 311 patients. There was no significant heterogeneity between studies (I^2^ = 31%; Q-test, P = 0.22), so Meta-analysis was performed using a fixed-effects model, which showed statistical significance between the two groups (Z = 2.36; P = 0.02; OR = -0.24; 95% CI (-0.43, -0.04)). It showed better pain symptom relief in the MC-PMMA group compared to the PMMA group at 6–12 months postoperatively (Fig 4).

#### 3.3.2 Comparison of postoperative ODI scores (≤3 days, 1–3 months, 6–12 months)

**Fig 5:**
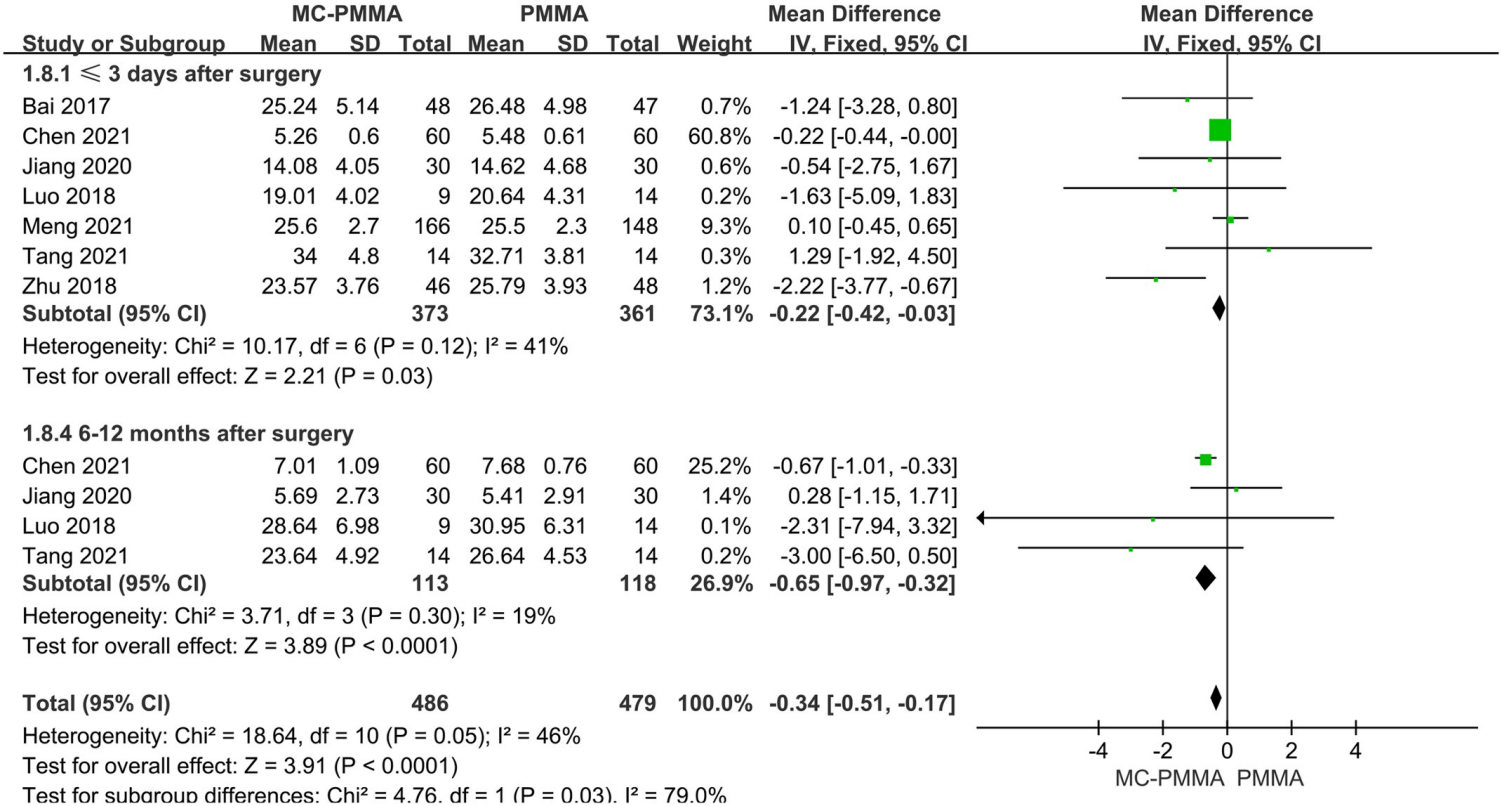
Forest plots of studies assessing ODI scores in the MC-PMMA vs. the PMMA group at postoperative (≤3 days, 6–12 months). The positive effect represents better postoperative functional recovery in patients with MC-PMMA bone cement after vertebroplasty, and the negative effect represents better postoperative functional recovery in patients with PMMA bone cement after vertebroplasty.

**Fig 6:**
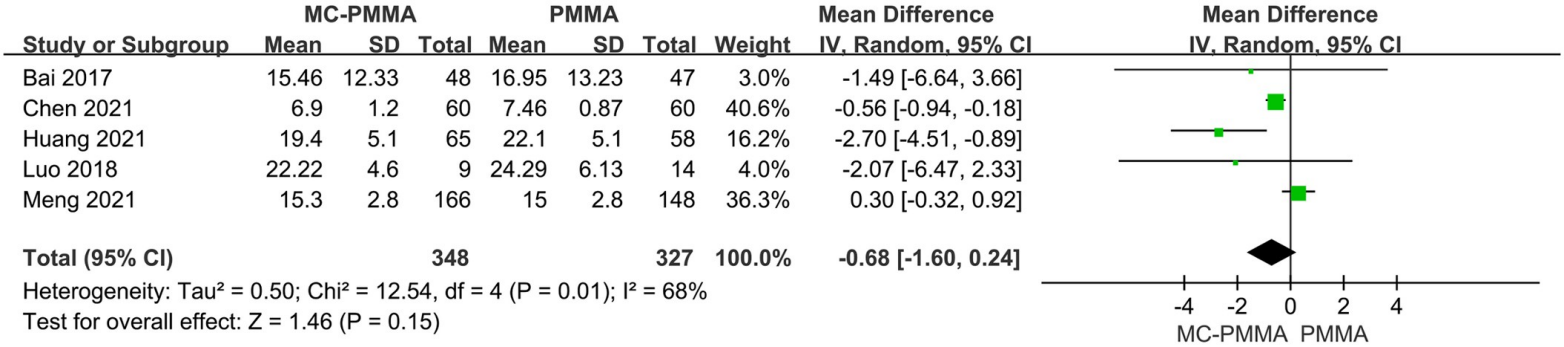
Forest plots of studies assessing ODI scores in the MC-PMMA vs. the PMMA group at postoperative (1–3 months).

Seven of the 12 studies reported comparisons of ODI scores within 3 days after surgery between the two groups, and a total of 734 patients were included. There was no significant heterogeneity among the studies (I^2^ = 41%; Q-test, P = 0.12). Therefore, Meta-analysis using a fixed-effects model showed statistically significant results (Z = 2.21; P = 0.03; OR = -0.22; 95% CI (-0.42, -0.03)) (Fig 5). Five studies reported comparisons of ODI scores at 1–3 months postoperatively between the two groups, enrolling a total of 675 patients. There was significant heterogeneity among the studies (I^2^ = 68%; Q-test, P = 0.01), and Meta-analysis using a random-effects model showed no statistical significance between the two groups (Z = 1.46; P = 0.15; OR = -0.68; 95% CI (-1.60, 0.24)) (Fig 6). Four studies reported comparisons of ODI scores at 6–12 months postoperatively between the two groups, enrolling a total of 231 patients. There was no significant heterogeneity among the studies (I^2^ = 19%; Q-test, P = 0.30), and the fixed-effects model was used to combine the effect sizes, which showed statistical significance between the two groups (Z = 3.89; P < 0.0001; OR = -0.65; 95% CI (-0.97, -0.32)). It showed that the MC-PMMA group had better functional recovery within 3 days postoperatively and at 6–12 months postoperatively compared with the PMMA group (Fig 5).

#### 3.3.3 Comparison of postoperative complications (adjacent vertebral fractures, bone cement leakage)

**Fig 7:**
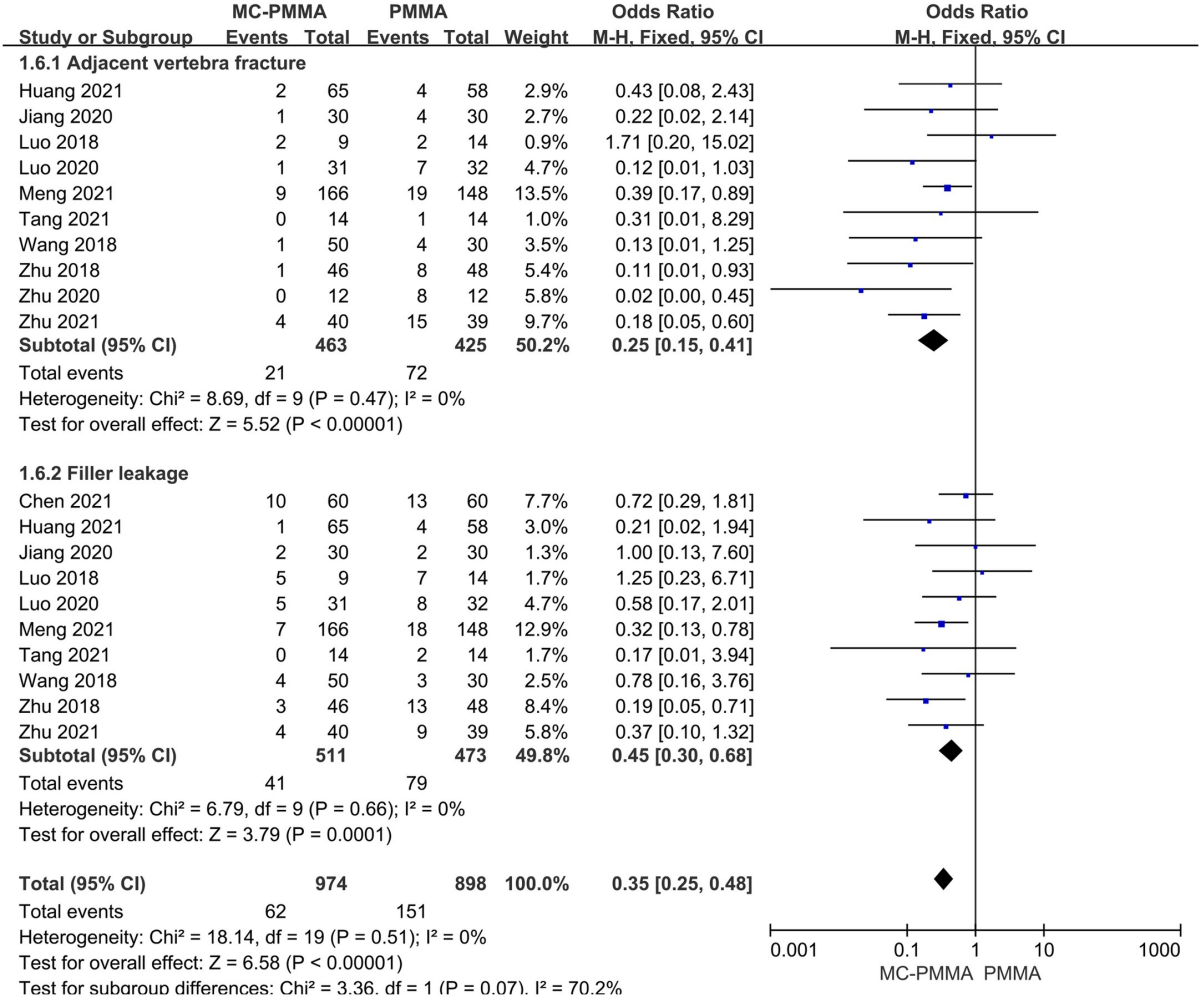
Forest plots of studies assessing postoperative complications (adjacent vertebral fractures, bone cement leakage) in the MC-PMMA vs. the PMMA group.

Ten of the 12 studies that reported a comparison of postoperative adjacent vertebral fracture rates between the two groups, which included a total of 888 patients. There was no heterogeneity among the studies by heterogeneity test (I^2^ = 0%; Q-test, P = 0.47), so Meta-analysis was performed using a fixed-effects model, and the results showed statistical significance (Z = 5.52; P < 0.00001; OR = 0.25; 95% CI (0.15, 0.41)) (Fig 7). Ten studies reported the rate of postoperative cement leakage between the two groups, enrolling a total of 984 patients. There was no heterogeneity among the studies (I^2^ = 0%; Q-test, P = 0.66), and Meta-analysis using a fixed-effects model showed statistical significance between the two groups (Z = 3.79; P = 0.0001; OR = 0.45; 95% CI (0.30, 0.68)) (Fig 7). It indicated that the incidence of postoperative adjacent vertebral fractures and cement leakage was lower in the MC-PMMA group compared with the PMMA group.

#### 3.3.4 Comparison of postoperative Cobb’s angle (<1 week, 3–6 months)

**Fig 8:**
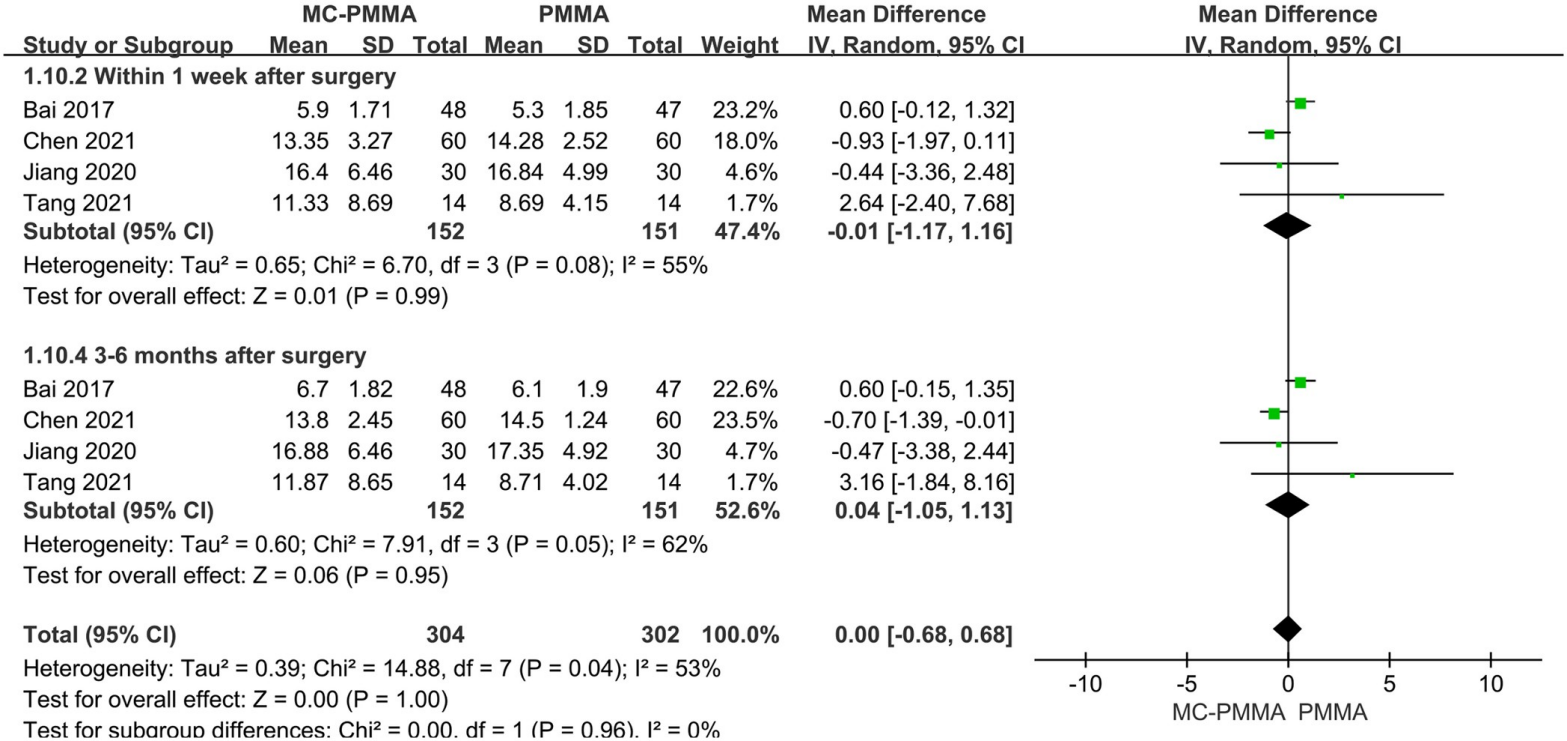
Forest plots of studies assessing Cobb’s angle in the MC-PMMA vs. the PMMA group at postoperative (<1 week, 3–6 months).

Four of the 12 studies reported a comparison of the Cobb angle of vertebral lordosis between the two groups within 1 week of surgery and included 303 patients in total. The results showed significant heterogeneity by the heterogeneity test (I^2^ = 55%; Q-test, P = 0.08), so Meta-analysis using a random-effects model showed no statistical significance between the two groups concerning the correction of the Cobb angle within 1 week postoperatively (Z = 0.01; P = 0.99; OR = -0.01; 95% CI (-1.17, 1.16)). Four studies reported vertebral kyphosis Cobb angle at 3–6 months postoperatively between the two groups, and a total of 303 patients were included. The results of the heterogeneity test showed significant heterogeneity (I^2^ = 62%; Q-test, P = 0.05), and Meta-analysis using a random-effects model showed no statistical significance in terms of correction of vertebral kyphosis Cobb angle at 3–6 months postoperatively between the two groups (Z = 0.06; P = 0.95; OR = 0.04; 95% CI (-1.05, 1.13)) (Fig 8).

#### 3.3.5 Comparison of postoperative CT values (1 year)

**Fig 9:**
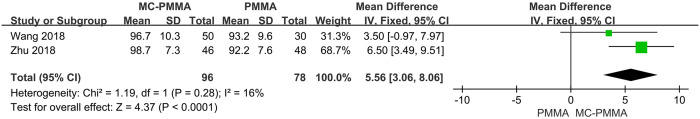
Forest plots of studies assessing CT values in the MC-PMMA vs. the PMMA group at postoperative (1 year).

Of the 12 studies, two reported a comparison of 1-year post-operative CT values between the two groups, including a total of 174 patients. The results of the heterogeneity test showed no significant heterogeneity between the studies (I^2^ = 16%; Q-test, P = 0.28), so Meta-analysis was performed using a fixed-effects model, and the results showed a statistically significant difference between the two groups (Z = 4.37; P < 0.0001; OR = 5.56; 95% CI (3.06, 8.06)) (Fig 9). It showed that the MC-PMMA group was more advantageous in the comparison of 1-year postoperative CT values compared with the PMMA group, i.e., the MC-PMMA group favored osteoclastogenesis and had a higher content of osteoblasts.

#### 3.3.6 Comparison of postoperative relative vertebral body anterior margin heights (≤1 week, 6–12 months)

**Fig 10:**
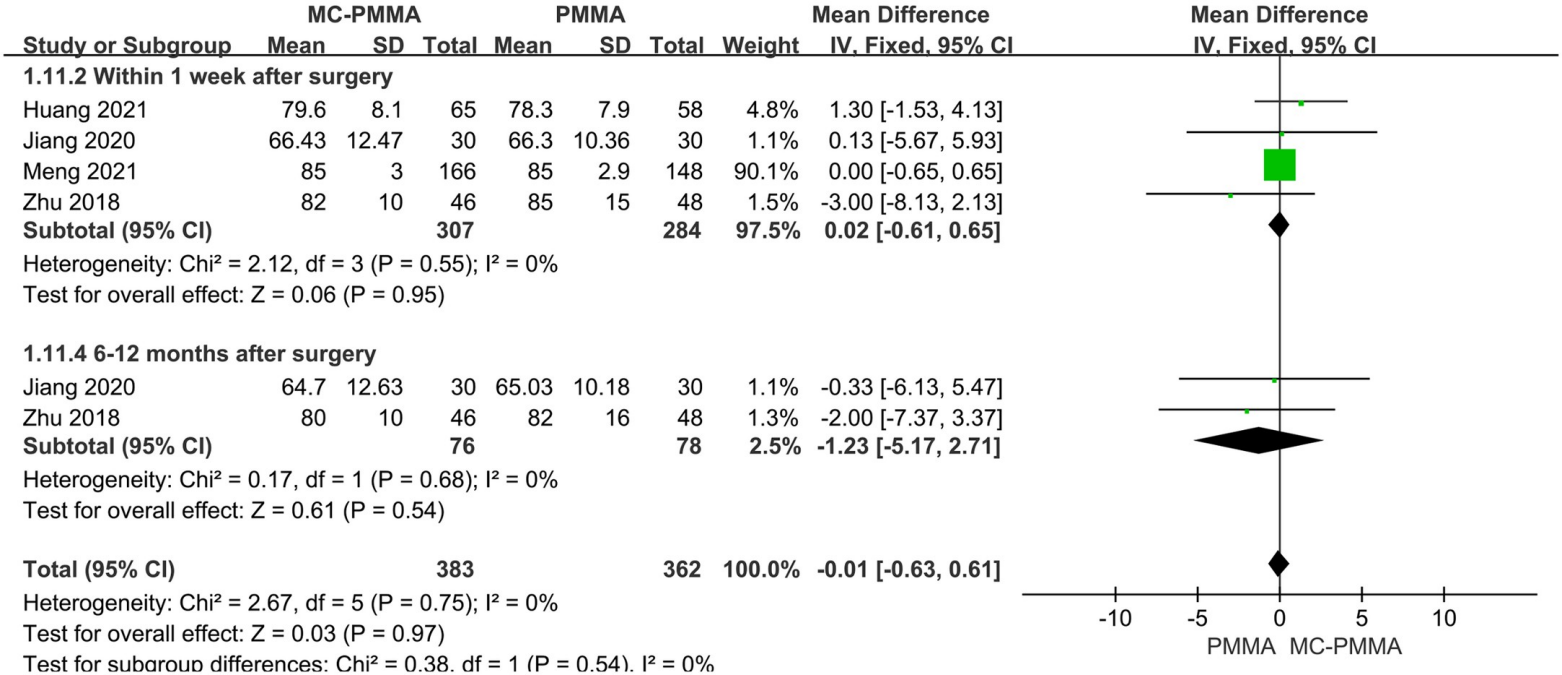
Forest plots of studies assessing relative vertebral body anterior margin heights in the MC-PMMA vs. the PMMA group at postoperative (≤1 week, 6–12 months).

Four of the 12 studies, which enrolled a total of 591 patients, reported a comparison of the relative height of the anterior vertebrae between the two groups at 1-week post-surgery. There was no heterogeneity between studies (I^2^ = 0%; Q-test, P = 0.55). Meta-analysis using a fixed-effects model showed no statistical significance between the two groups (Z = 0.06; P = 0.95; OR = 0.02; 95% CI (-0.61, 0.65)). Two studies, including 154 patients, reported a comparison of the relative height of the front margin of the vertebrae between the two groups at 6–12 months after surgery. There was no heterogeneity between the studies (I^2^ = 0%; Q-test, P = 0.68), and Meta-analysis using a fixed-effects model showed no statistical significance between the two groups (Z = 0.61; P = 0.54; OR = -1.23; 95% CI (-5.17, 2.71)) (Fig 10).

#### 3.3.7 Comparison of intraoperative bone cement filling volume

**Fig 11:**
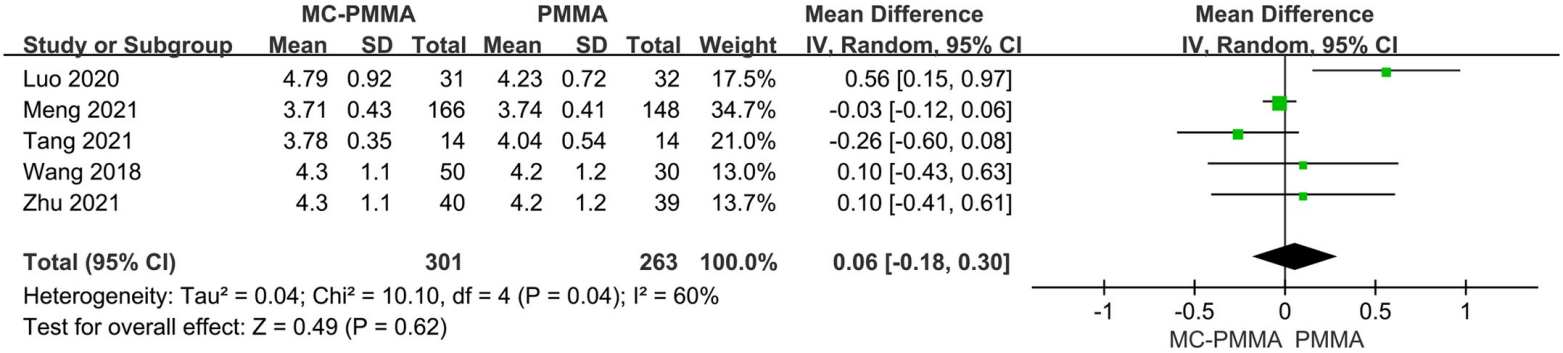
Forest plots of studies assessing intraoperative bone cement filling volume in the MC-PMMA vs. the PMMA group.

Five of the 12 studies reported intraoperative bone cement filling volume comparisons between the two groups, enrolling a total of 564 patients. The results of the heterogeneity test showed significant heterogeneity among the studies (I^2^ = 60%; Q-test, P = 0.04), and Meta-analysis using a random-effects model showed that there was no statistically significant difference between the two groups in terms of the amount of intraoperative bone cement filled (Z = 0.49; P = 0.62; OR = 0.06; 95% CI (-0.18, 0.30)) (Fig 11).

#### 3.3.8 Comparison of intraoperative bone cement filling times

**Fig 12:**
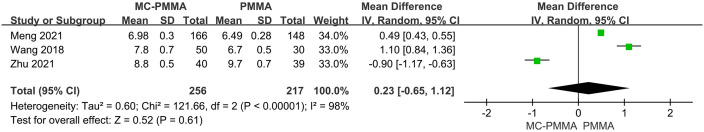
Forest plots of studies assessing intraoperative bone cement filling times in the MC-PMMA vs. the PMMA group.

Three of the 12 studies reported a comparison of intraoperative bone cement filling time between the two groups, enrolling a total of 473 patients. There was significant heterogeneity among the studies (I^2^ = 98%; Q-test, P < 0.00001), and Meta-analysis using a random-effects model showed no statistically significant difference between the two groups in terms of intraoperative bone cement filling time (Z = 0.52; P = 0.61; OR = 0.23; 95% CI (-0.65, 1.12)) (Fig 12).

#### 3.3.9 Comparison of operation times

**Fig 13:**
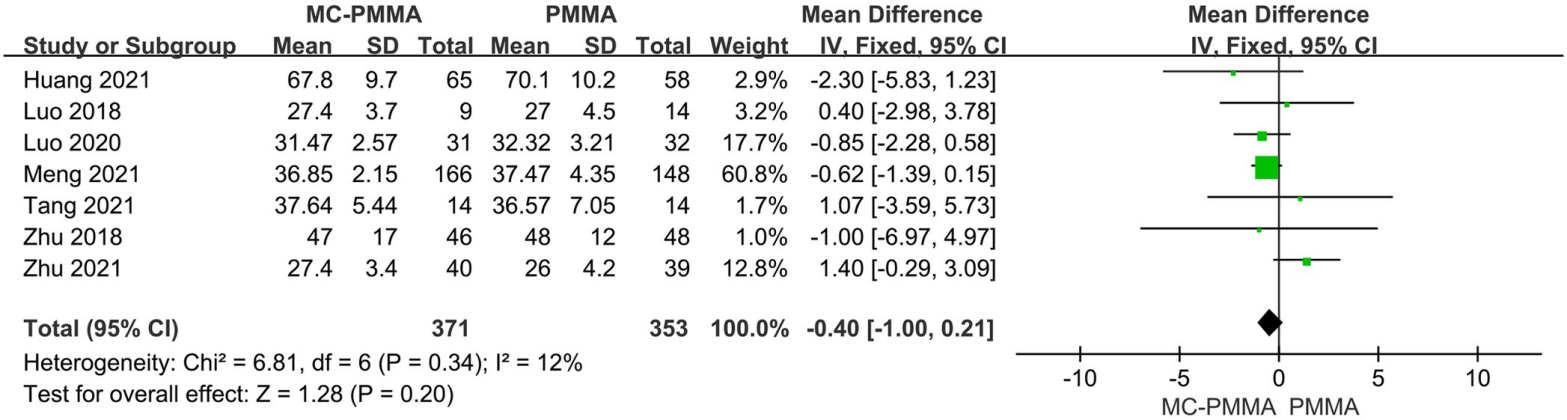
Forest plots of studies assessing operation times in the MC-PMMA vs. the PMMA group.

Seven of the 12 studies reported comparisons of operative time between the two groups, enrolling a total of 724 patients. There was no significant heterogeneity among the studies (I^2^ = 12%; Q-test, P = 0.34), and Meta-analysis using a fixed-effects model showed that there was no statistically significant difference between the two groups in terms of operative time (Z = 1.28; P = 0.20; OR = -0.40; 95% CI (-1.00, 0.21)) (Fig 13).

#### 3.3.10 Comparison of intraoperative bleeding volumes

**Fig 14:**
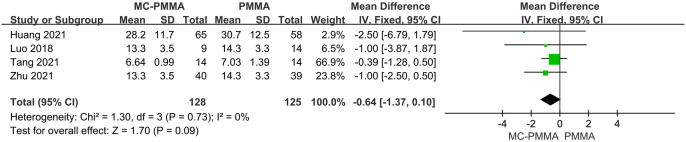
Forest plots of studies assessing intraoperative bleeding volumes in the MC-PMMA vs. the PMMA group.

Of the 12 studies, four reported intraoperative bleeding comparisons between the two groups, enrolling 253 patients in total. There was no heterogeneity among the studies (I^2^ = 0%; Q-test, P = 0.73), and Meta-analysis using a fixed-effects model showed no statistically significant difference between the two groups in terms of intraoperative bleeding (Z = 1.70; P = 0.09; OR = -0.64; 95% CI (-1.37, 0.10)) (Fig 14).

#### 3.3.11 Comparison of hospital length of stay

**Fig 15:**
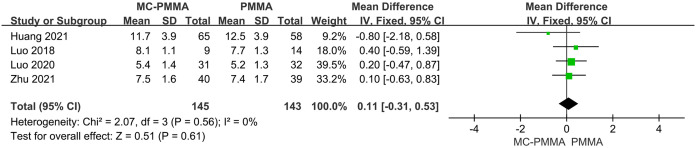
Forest plots of studies assessing hospital length of stay in the MC-PMMA vs. the PMMA group.

Four of the 12 studies reported a comparison of length of stay between the two groups, enrolling a total of 288 patients. There was no heterogeneity among the studies (I^2^ = 0%; Q-test, P = 0.56), and a fixed-effects model was used to combine the effect sizes, which showed no statistical significance between the two groups in terms of length of hospitalization (Z = 0.51; P = 0.61; OR = 0.11; 95% CI (-0.31, 0.53)) (Fig 15).

#### 3.3.12 Publication bias and sensitivity analysis

**Fig 16:**
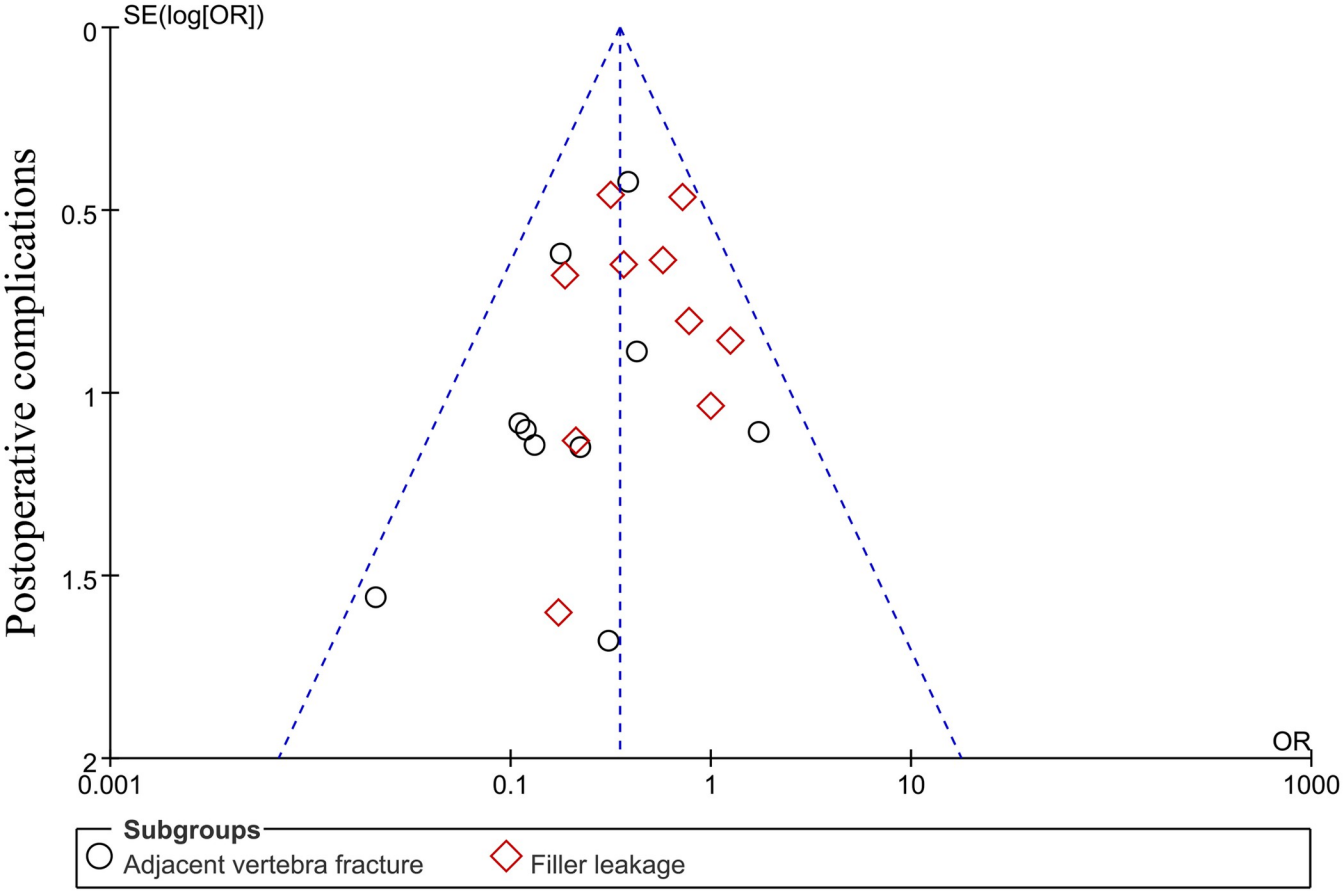
Funnel plots of postoperative complications (adjacent vertebral fractures, bone cement leakage).

Review Manager 5.4 Statistical software provided by the Cochrane Collaboration was used to detect publication bias for outcome indicators with ≥ 10 studies. Therefore, publication bias analysis was performed for postoperative complications (adjacent vertebral fracture, cement leakage). The results of the funnel plot showed that the points were symmetrically distributed on both sides of the vertical dashed line indicating the amount of combined effect, and none of the points were outside the diagonal dashed line indicating the 95% CI, suggesting that there was no obvious publication bias (Fig 16), and the data of this study were considered to be relatively stable and reliable by sensitivity analysis.

## 4. Discussion

Vertebral compression fractures are one of the most common types of fractures in patients with osteoporosis and are commonly seen in the elderly, mostly presenting as wedge-shaped changes in the vertebral body, resulting in pain at the fracture site, loss of vertebral height, spinal instability, and can even lead to kyphotic deformity [25]. Vertebroplasty, represented by PVP and PKP, is a minimally invasive technique in which a working channel is inserted through the pedicle or paravertebral safety area to reach the injured vertebral after the body surface is positioned, and bone cement and other fillers are injected to treat vertebral compression fractures and other diseases to reduce pain and strengthen the damaged vertebral body [26]. It has the advantages of less trauma, early stabilization of the fracture, effective pain relief, promotion of rapid patient recovery, avoidance of long-term bed rest and other related complications, and significant improvement in patient quality of life [27]. Bone cement is a kind of biomaterial with self-coagulation characteristics, which can be filled between bone and inner plants and potential bone space to play a specific role, which can ensure the real-time stability of the injured vertebral body after implantation, which is conducive to early functional exercise after surgery, and then obtain good clinical efficacy [28]. Currently, PMMA bone cement is the most widely used bone cement in vertebroplasty because of its advantages of significantly improving the strength of the vertebral body and rapidly relieving pain [6]. In contrast, MC-PMMA bone cement is based on PMMA bone cement by incorporating MC particles to down-regulate the elastic modulus of the cured body of bone cement on the premise of possessing the physical strength of PMMA bone cement curing; meanwhile, its biological inertia is improved so that it can be degraded and absorbed in vivo and has good osteogenic activity [18, 29]. The number of studies comparing the clinical efficacy of PMMA and MC-PMMA bone cement in vertebroplasty is increasing, and here we compare the clinical efficacy and safety of the two filling materials by meta-analysis.

The primary purpose of vertebroplasty is to relieve pain symptoms and maintain spinal stability for good functional recovery and clinical efficacy. The postoperative pain VAS score and the ODI score are commonly used to assess patient pain relief and postoperative functional recovery. The results of Meta-analysis showed that the MC-PMMA group had an advantage in postoperative (6–12 months) pain VAS scores and postoperative (≤3 days, 6–12 months) ODI scores, which was consistent with the findings of Luo et al. [14] and Zhu et al. [18]. Combined with the analysis of the postoperative pain VAS score and ODI score results, we believe that it is reasonable to conclude that the MC-PMMA group has better postoperative clinical outcomes in the distant postoperative period (>6 months). Meanwhile, our comparison of the CT values 1 year after surgery showed that the CT values were higher in the MC-PMMA group, which was consistent with the results of Zhu et al. [17], i.e., it indicated that the proliferation of osteoblasts in the vertebral body was more pronounced in the MC-PMMA group, and it also indicated that the MC-PMMA group had better osseointegration characteristics; however, the inclusion of literature in the current meta-analysis is limited, so the adoption of this result should be done with caution. Studies have shown that after gradual degradation and resorption of MC particles, MC-PMMA bone cement continuously promotes the migration of osteoblasts and guides the growth of new bone tissues to the porous structure, which results in the formation of a staggered bone mosaic between the cement and the bone tissues at the implantation site, and also indicates that MC-PMMA bone cement has no obvious cytotoxicity and can promote cell proliferation. Moreover, Zhu et al. [18] demonstrated through in vivo animal experiments that MC-PMMA bone cement can promote the differentiation of bone mesenchymal stem cells (BMSCs), increase the activity of alkaline phosphatase (ALP) (a marker of osteogenic differentiation), and thus can promote bone repair. Combined with the postoperative pain VAS score, postoperative ODI score, and 1-year postoperative CT value, it is reasonable to conclude that the MC-PMMA bone cement group, due to its biodegradability and bioactivity, has better integration ability with the neighboring vertebral bodies in the long-term postoperative period (>6 months) and thus has better pain symptom relief and postoperative functional recovery in the long-term postoperative period.

Cement leakage and adjacent vertebral fractures are critical safety issues in vertebroplasty, and adjacent vertebral fractures and cement leakage into the personal or spinal canal may lead to serious consequences. It has been suggested that cement leakage is related to factors such as the amount of cement filling, cement viscosity, and the degree of vertebral injury [30, 31]. Cement viscosity and degree of vertebral body injury were not graded in the literature included in this study, so they were not analyzed. The results of the meta-analysis showed that MC-PMMA bone cement was significantly better than PMMA bone cement in reducing the rate of cement leakage, and there was no statistically significant difference between the two groups of filling materials in terms of the amount of cement filling. It indicated that the MC-PMMA bone cement group had a lower incidence of bone cement leakage without increasing the risk factor of bone cement filling volume. This result is consistent with the findings of Luo et al. [14], who concluded that a lower rate of cement leakage is more favorable for elderly patients with vertebral compression fractures, avoiding other complications associated with free cement fragmentation and contributing to postoperative functional recovery. In addition, the MC-PMMA group was significantly lower than the PMMA group in the comparison of the incidence of adjacent vertebral fractures. This result is consistent with the findings of Luo et al. [14], Wang et al. [16] and Zhu et al. [18]. Wang et al. [16] concluded that the excessive elastic modulus of PMMA bone cement curing the body was the most important cause of vertebral bone wear or even fracture of adjacent vertebrae after vertebroplasty. And Shen et al. [32] concluded that the cement disc leakage accelerated the degenerative changes of the intervertebral disc, which decreased the role of the intervertebral disc in cushioning the undesirable stresses and made the stress distribution uneven, and the intervertebral disc tissues and the vertebral body after vertebroplasty formed a high-stiffness segment together, which increased the adjacent plate stresses and induced the fracture of the adjacent vertebral body. Combined with the analysis of the above results, it was concluded that the MC-PMMA group had a significant advantage in reducing postoperative complications (adjacent vertebral body fracture, cement leakage), and it was also shown that the MC-PMMA bone cement was able to reduce the modulus of elasticity of the curing body while maintaining the rigidity and strength required for vertebral body stability so that it was close to that of the autogenous bone, which was conducive to promoting postoperative efficacy and improving the quality of life.

The results of this meta-analysis showed that there was no statistical significance in the postoperative vertebral body posterior convexity Cobb angle (≤1 week, 3–6 months) and relative vertebral body anterior margin height (≤1 week, 6–12 months). It indicates that PMMA bone cement modified by MC has both the properties of PMMA bone cement, which can effectively maintain the height of the injured vertebrae and maintain body posture without shortening the use time of the cement. And there is no statistical significance between the two groups of filling materials in terms of the operation time, intraoperative bleeding, cement filling time, and hospitalization time, then it can be shown that the modification of PMMA by the addition of MC can be sufficiently mixed between the two, which has little effect on the injectability and maneuverability of the original PMMA bone cement, and therefore does not increase the additional surgical burden and intraoperative injuries so that the MC-PMMA bone cement has a practical clinical application.

This meta-analysis comes with its limitations: 1. There are differences in the properties of MC-PMMA bone cement and clinical effects for different proportions of added MC. 2. Most of the literature included in this study is from the Asian population, which is not broadly representative and has publication bias. 3. A total of 12 articles have been included in the literature, and there are fewer RCTs with lower levels of evidence, so the results need to be adopted with caution. 4. The adopted data is limited to allow for a wider range of comparisons and subgroup analyses. In the future, the conclusions derived from this study will be better validated and supported as the sample size of the study increases, the follow-up time is extended, and the quality of the sample size included is improved.

## 5. Conclusion

In conclusion, MC-PMMA bone cement has significant advantages in reducing postoperative complications (adjacent vertebral fractures, bone cement leakage) and in the relief of pain symptoms and functional recovery in the long term (>6 months) after surgery. MC-PMMA bone cement combines the advantages of PMMA bone cement with its clinical handling characteristics and sufficient curing mechanical strength, while reducing the elastic modulus of the cured body and giving it better biocompatibility, thus reducing the corresponding postoperative complications and enabling the bone cement to form osseointegration with the patient’s own bone tissue by inducing osteoclastogenesis, so that it can exist stably and firmly in the implanted vertebral body, maintaining spinal stability and obtaining better pain relief, thus obtaining better clinical efficacy.

## Supporting information

S1 Table The data of meta-analysis. (XLS)

S1 Checklist PRIMSA abstract checklist. (DOCX)
